# Clec14a genetically interacts with Etv2 and Vegf signaling during vasculogenesis and angiogenesis in zebrafish

**DOI:** 10.1186/s12861-019-0188-6

**Published:** 2019-04-05

**Authors:** Karolina Pociute, Jennifer A. Schumacher, Saulius Sumanas

**Affiliations:** 1 Division of Developmental Biology, Cincinnati Children’s Hospital Medical Center, 3333 Burnet Ave, Cincinnati, OH 45229 USA; 2 Present Address: Vilnius University Life Sciences Center, Sauletekio 7, 10223 Vilnius, Lithuania; 3 Department of Pediatrics, University of Cincinnati College of Medicine, 3333 Burnet Ave, Cincinnati, OH 45229 USA

**Keywords:** Clec14, Zebrafish, Angiogenesis, Vasculogenesis, Vascular endothelial, Vegf, etv2, Etsrp

## Abstract

**Background:**

C-lectin family 14 Member A (Clec14a) is a transmembrane protein specifically expressed in vascular endothelial cells during embryogenesis. Previous in vitro and in vivo studies have provided conflicting data regarding Clec14a role in promoting or inhibiting angiogenesis, therefore its functional role in vascular development remains poorly understood.

**Results:**

Here we have generated a novel *clec14a* mutant allele in zebrafish embryos using TALEN genome editing. *clec14a* mutant embryos exhibit partial defects and delay in the sprouting of intersegmental vessels. These defects in angiogenesis are greatly increased upon the knockdown of a structurally related C1qr protein. Furthermore, a partial knockdown of an ETS transcription factor Etv2 results in a synergistic effect with the *clec14a* mutation and inhibits expression of early vascular markers in endothelial progenitor cells, arguing that *clec14a* is involved in promoting vasculogenesis. In addition, Clec14a genetically interacts with Vegfa signaling. A partial knockdown of Vegfaa function in the *clec14a* mutant background resulted in a synergistic inhibition of intersegmental vessel sprouting.

**Conclusions:**

These results argue that *clec14a* is involved in both vasculogenesis and angiogenesis, and suggest that Clec14a genetically interacts with Etv2 and Vegf signaling during vascular development in zebrafish embryos.

**Electronic supplementary material:**

The online version of this article (10.1186/s12861-019-0188-6) contains supplementary material, which is available to authorized users.

## Background

New blood vessels form by two distinct mechanisms, vasculogenesis and angiogenesis. Vasculogenesis involves formation of new blood vessels through differentiation of vascular endothelial cells de novo, while angiogenesis involves growth of new vasculature through sprouting from the existing blood vessels [1]. Despite a significant progress in identifying signaling pathways that regulate vascular development, the molecular mechanisms that regulate vasculogenesis and angiogenesis are still only partially understood.

C-type lectin family 14 member A (Clec14a, also known as C1qrl, Crl) is a transmembrane protein which contains a C-type lectin and EGF-like domains [2]. Its protein sequence is highly conserved between multiple vertebrates including zebrafish, mouse and humans. We have previously described its expression in vascular endothelial cells in zebrafish embryos [3]. Expression of *clec14a* was greatly downregulated in *cloche / npas4l* mutants, deficient in hematopoietic and vascular development. Similar to zebrafish, *Clec14a* is specifically expressed in vascular endothelial cells in mouse embryos and human tissues, and its expression is greatly upregulated during tumor angiogenesis [2, 4]. In vitro studies have demonstrated that CLEC14A promotes filopodia formation, cell migration and tubulogenesis [2, 4]. In zebrafish, it has been reported that Clec14a functions redundantly with a related protein C1qr / Cd93 in promoting angiogenesis [5]. Double *clec14a* and *c1qr* mutant embryos showed greatly inhibited angiogenesis and reduced *cadherin 5 (cdh5)* expression, which could be rescued by synthetic *cdh5* mRNA injection [5]. In contrast, mouse *Clec14a* mutants displayed increased angiogenesis and lymphangiogenesis, accompanied by an increase in hemorrhages and vessel dilations [6]. *Clec14a* deficiency resulted in reduced endothelial expression of *Vascular Endothelial Growth Factor Receptor 3 (Vegfr3),* while expression of *Vegfr2* was increased. In addition, Clec14a was shown to physically interact with Vegfr3 [6].

While all previous studies point to the role of Clec14a in regulating angiogenesis, it is currently unclear why the *Clec14a* knockout in mouse embryos results in increased angiogenesis, while the zebrafish *clec14a* mutants show reduced angiogenesis, similar to the CLEC14A knockdown in cell culture. Furthermore, it is currently unknown if Clec14a plays any role in vasculogenesis, in addition to its previously reported role in angiogenesis. To address these questions, we generated a novel zebrafish *clec14a* mutant allele using transcription activator-like effector nucleases (TALEN)- mediated genome editing. Our results show that *clec14a* mutants display subtle defects in angiogenic sprouting which are greatly increased upon functional inhibition of a related C1qr protein. We demonstrate that *clec14a* genetically interacts with ETS transcription factor *etv2* during vasculogenesis, demonstrating its novel role in promoting differentiation of vascular endothelial progenitors. We also show a synergistic genetic interaction between *clec14a* and Vegf signaling.

## Results

**Fig. 1:**
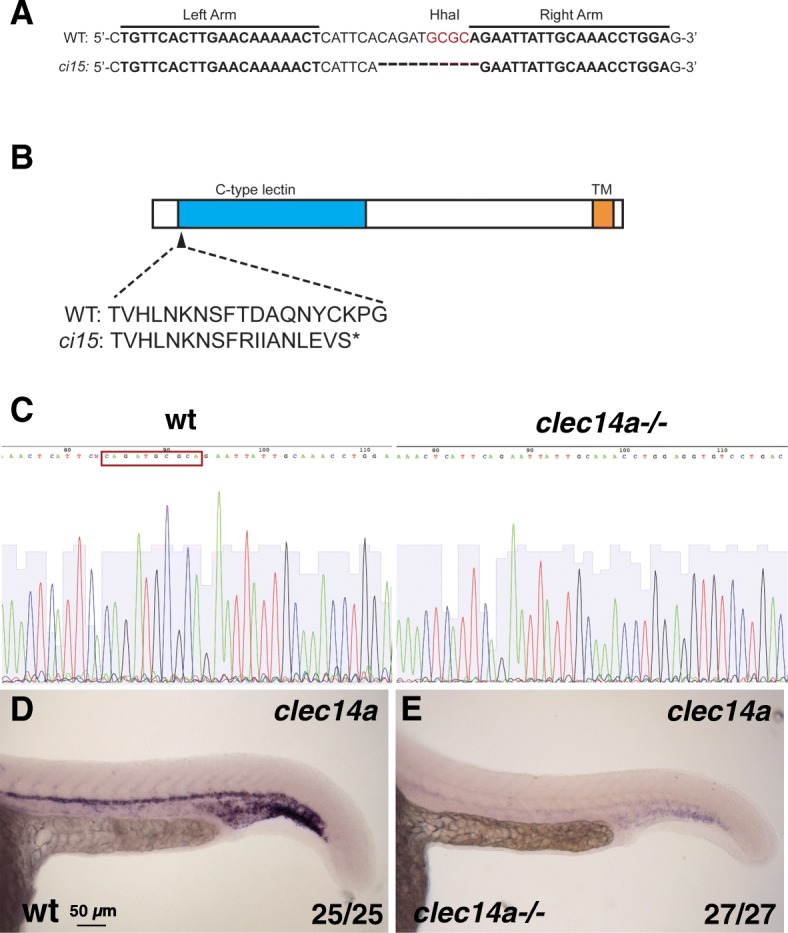
Generation of *clec14a* mutants by TALEN-mediated genome editing. **a** Wild-type and *clec14a*^*ci15*^ mutant sequence showing the binding regions for TALE nucleases and the 10 bp deletion in the *clec14a* mutants which includes the deletion of HhaI restriction site used for genotyping. **b** A diagram of Clec14a protein sequence which includes C-type lectin and transmembrane (TM) domains. *clec14a*^*ci15*^ mutation is predicted to cause a frameshift early in the protein coding sequence starting at the amino acid 44 and would lead to a premature stop codon. **c**, **d** DNA sequencing chromatogram shows a 10 bp deletion (boxed-in wild-type sequence) in the cDNA of *clec14a* mutants. **d**, **e** In situ hybridization analysis for *clec14a* expression in wt embryos (**d**) and *clec14a* mutants (**e**) at 24 hpf. Note a significant reduction of *clec14a* expression in *clec14a* mutant embryos. The experiment has been replicated twice; the combined number of embryos analyzed and showing the phenotype is shown in the lower right corner

To analyze the function of zebrafish *clec14a* during vascular development, we generated *clec14a* mutant allele using TALEN genome editing [7]. The *clec14a*^*ci15*^ mutant allele carries a 10 bp deletion and is predicted to result in a frameshift and premature stop codon early in the open reading frame at amino acid position 44 (Fig. 1a, b). DNA sequencing of *clec14a* coding sequence amplified by PCR from cDNA obtained from *clec14a* mutant embryos at 24 hpf confirmed the presence of expected 10 bp deletion (Fig. 1c, d). No other splice variants or additional PCR bands were identified in *clec14a* cDNA of *clec14a* mutant embryos (data not shown). Expression of *clec14a* was greatly reduced in *clec14a* homozygous mutant embryos as analyzed by in situ hybridization (ISH) (Fig. 1c, d). These data argue that the level of *clec14a* mRNA is greatly reduced in *clec14a* mutants, and the remaining transcript does not code for a functional protein, suggesting that the mutation allele is null or close to null. Nevertheless, homozygous *clec14a* mutant embryos were morphologically normal, did not show any obvious defects and were viable as adults (data not shown).

**Fig. 2:**
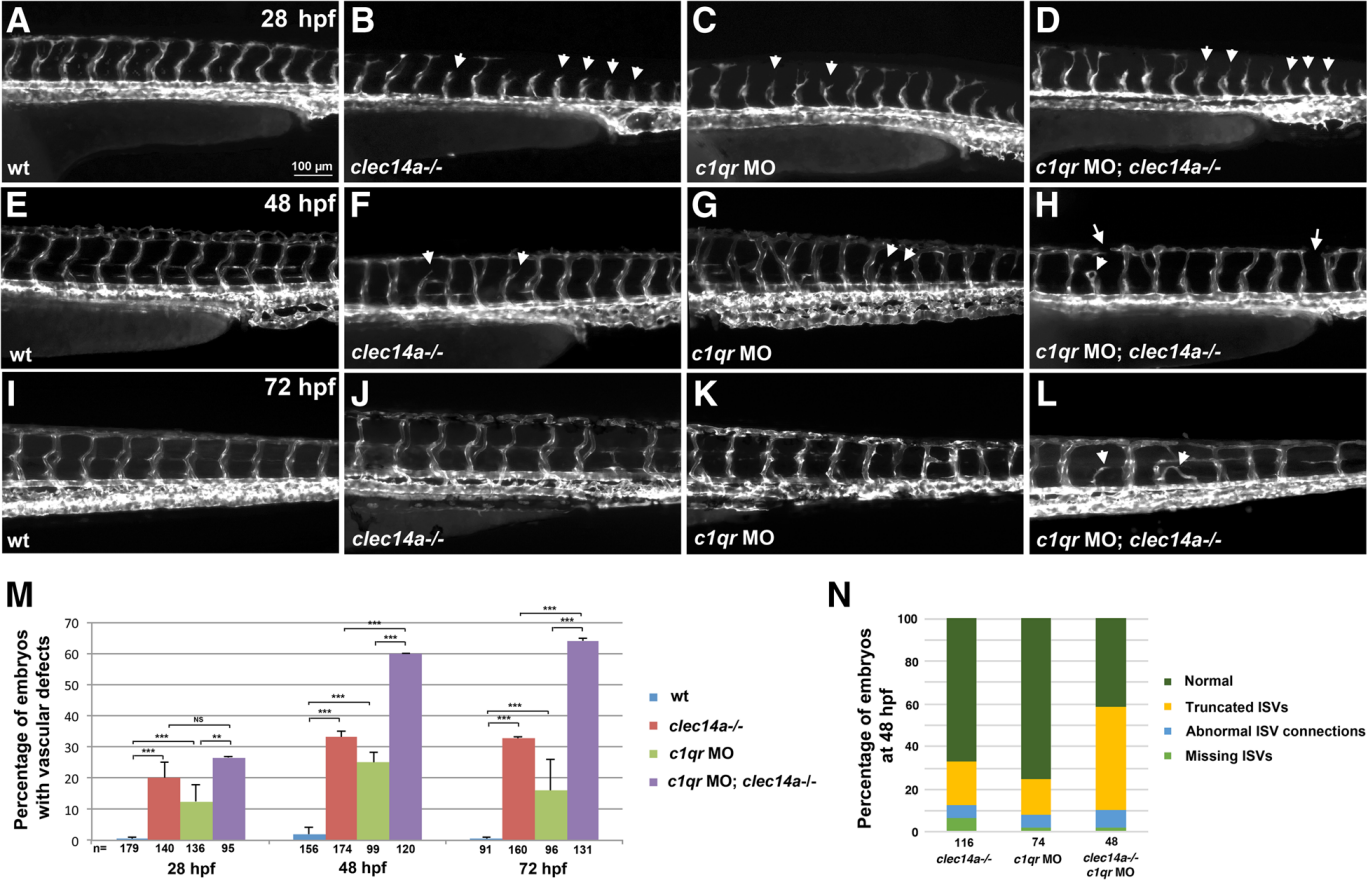
Clec14a and C1qr have a partially redundant function during angiogenic sprouting. *Kdrl: GFP* expression in the trunk region imaged by fluorescent microscopy at 28 hpf (**a**-**d**), 48 hpf (**e**-**h**) and 72 hpf (**i**-**l**). **a**, **e**, **i** Wild-type control embryos; **b**, **f**, **j** *clec14a−/−* mutants; **c**, **g**, **k** Wild-type embryos injected with 10 ng of *c1qr* MO; **d**, **h**, **l** *clec14a−/−* mutant embryos injected with 10 ng of *c1qr* MO. Note the delayed intersegmental vessel sprouting in *clec14a* mutants (**b**) and *c1qr* MO embryos (**c**) at 24 hpf (arrowheads). Partial sprouts (arrowheads) and abnormal vascular connections (arrows) are apparent in the *clec14a−/−; c1qr* MO embryos (**d**, **h**, **l**). **m** Percentage of embryos with vascular defects. ** *p* < 0.01; *** *p* < 0.001; NS, no significance, Fisher’s exact test. Data were combined from 3 independent experiments. Error bars show standard error. **n** Distribution of different types of phenotypes in embryos at 48 hpf. Data were combined from two independent experiments

A previous study suggested that Clec14a functions redundantly with a related protein C1qr [5]. Recent studies have shown that expression of functional homologs is often upregulated in genetic mutants as a part of a compensatory mechanism [8]. Indeed, *c1qr* expression was increased by 2.9-fold (±0.7 SEM) in *clec14a−/−* mutant embryos at 24 hpf by qPCR analysis compared to wild-type embryos (*p* = 0.03, Student’s t-test). To test if *c1qr* compensates for the loss of *clec14a* function, we designed and injected a translation-blocking morpholino (MO) against C1qr protein in either wild-type or *clec14a−/−* embryos crossed into vascular endothelial specific *kdrl: GFP* transgenic background. Phenotypic analysis revealed that about 20% of *clec14a−/−* mutants and 12.5% of *c1qr* MO injected embryos showed reduced or delayed intersegmental vessel (ISV) sprouting at 28 hpf. This percentage was slightly higher (26.5%) in double knockdown *c1qr* MO; *clec14a−/−* embryos, which was not a statistically significant difference (Fig. 2a-d, m). Sprouting defects including truncated or missing ISVs and abnormal ISV connections persisted in a significant fraction (16–33%) of *clec14a−/−* or *c1qr* MO injected embryos at 48 and 72 hpf (Fig. 2e-n). In contrast, the majority of double knockdown *c1qr* MO; *clec14a−/−* embryos (60–64%) displayed abnormal ISV connections, mispatterned ISVs, partial ISV sprouts and defects in the formation of the dorsal longitudinal anastomotic vessel (DLAV) (Fig. 2h, l-n). Overall, the ISV sprouting defects observed in *c1qr* MO; *clec14a−/−* embryos were similar to the previously reported defects in *c1qr; clec14a* double mutants [5], arguing that the MO specifically inhibits C1qr function. To confirm that *c1qr MO* effectively inhibits protein expression, we designed a GFP reporter by fusing 939 bp of *c1qr* genomic sequence immediately upstream of the translation-initiating ATG codon to the GFP reporter and SV40 polyA sequence. 11% of embryos injected with this construct showed GFP expression in multiple cells in the trunk and tail region while none of the embryos co-injected with the GFP reporter and *c1qr* MO showed such expression (Additional file 1: Figure S1). This argues that *c1qr* MO can effectively inhibit GFP reporter expression. Injection of a control MO or a 5-base mismatch MO into *clec14a* mutants did not affect the percentage of embryos with ISV defects, further arguing for the specificity of the observed phenotype (Additional file 1: Figures S2 and S3). Thus, C1qr and Clec14a function partially redundantly during angiogenic sprouting.

**Fig. 3:**
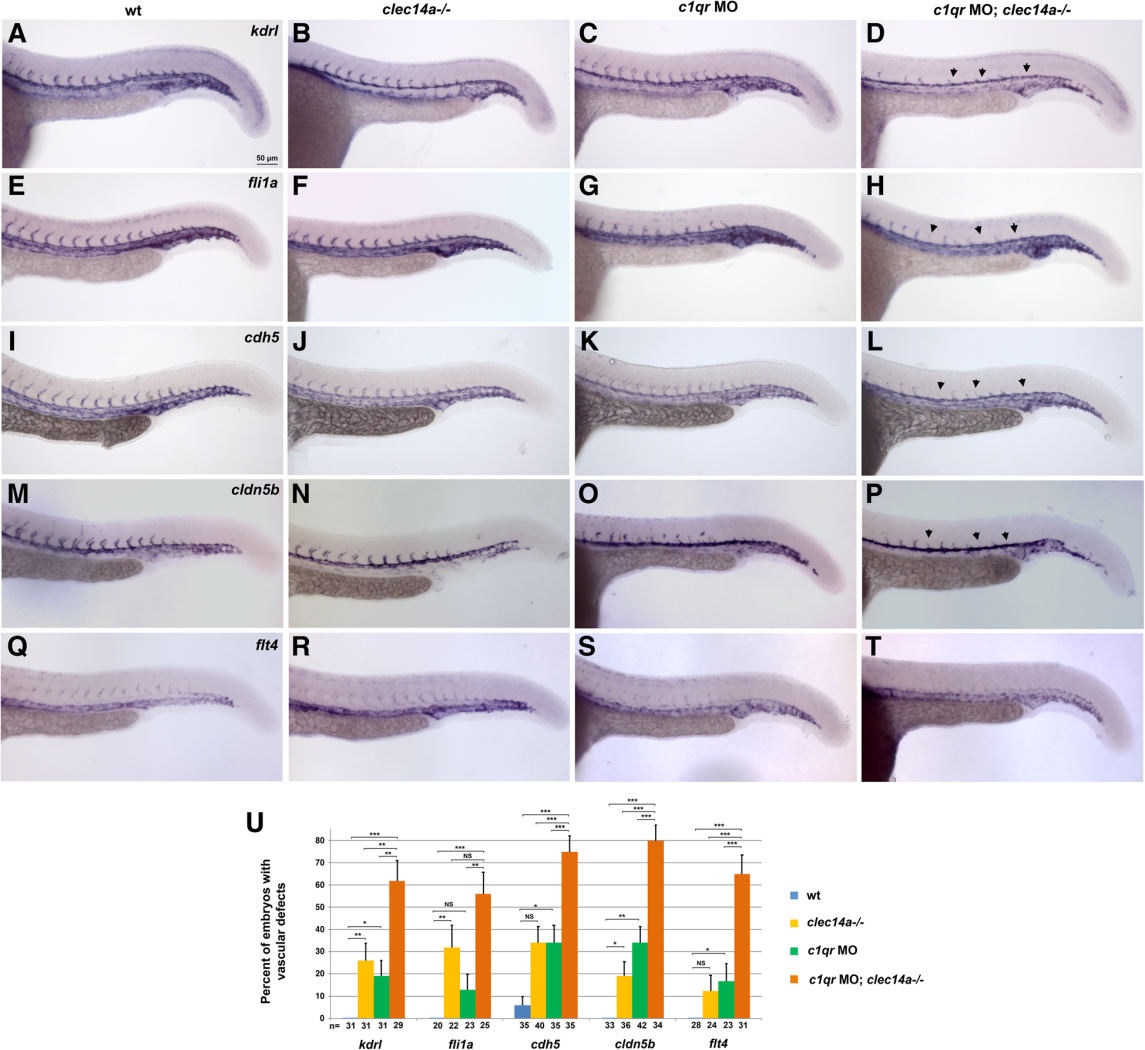
Analysis of vascular marker expression by in situ hybridization at 24 hpf in *clec14a−/−* embryos injected with 10 ng of *c1qr* MO. **a**-**d** *kdrl,* **e**-**h** *fli1a,* **i**-**l** *cdh5,* **m**-**p** arterial marker *cldn5b,* **q**-**t** venous marker *flt4* expression. Note the strong inhibition of angiogenic sprouting in the double *clec14a−/−*; *c1qr* MO embryos (arrowheads). Only weak or partial inhibition of sprouting is observed *in clec14a−/−* or *c1qr* MO embryos. **u** Percentage of embryos with defects in ISV sprouting is greatly increased among *c1qr* MO; *clec14a−/−* embryos compared to *c1qr* MO or *clec14a−/−* embryos. n refers to the number of embryos analyzed for each marker. *p < 0.05; ** *p* < 0.01; *** *p* < 0.001; NS, no significance, Fisher’s exact test. Data were combined from two independent experiments. Error bars show standard error

We then analyzed expression of vascular markers *in clec14a*−/− mutants, *c1qr* MO-injected embryos and double *clec14a−/−; c1qr* MO embryos using in situ hybridization (ISH). The majority of *clec14a−/−* or *c1qr* MO embryos displayed normal expression of Vegfr2 homolog *kdrl,* ETS transcription factor *fli1a* and *VE-cadherin / cdh5,* while a fraction of these embryos (13–34%) exhibited inhibition of ISV sprouting (Fig. 3a-c, e-g, i-k, u, and data not shown). The percentage of affected embryos correlated closely to the percentage of embryos showing ISV defects based on *kdrl: GFP* fluorescence analysis. In contrast, double *c1qrMO; clec14a−/−* embryos showed strong reduction in ISV sprouting (Fig. 3d, h, l, u). Expression of the arterial marker *cldn5b* in the dorsal aorta (DA) and venous marker *flt4* in the posterior cardinal vein (PCV) was not significantly affected in *clec14a−/−, c1qrMO* or double knockdown embryos, suggesting that arteriovenous patterning was not affected while ISV sprouting was partially inhibited, similar to the results obtained with the other markers (Fig. 3m-u).

**Fig. 4:**
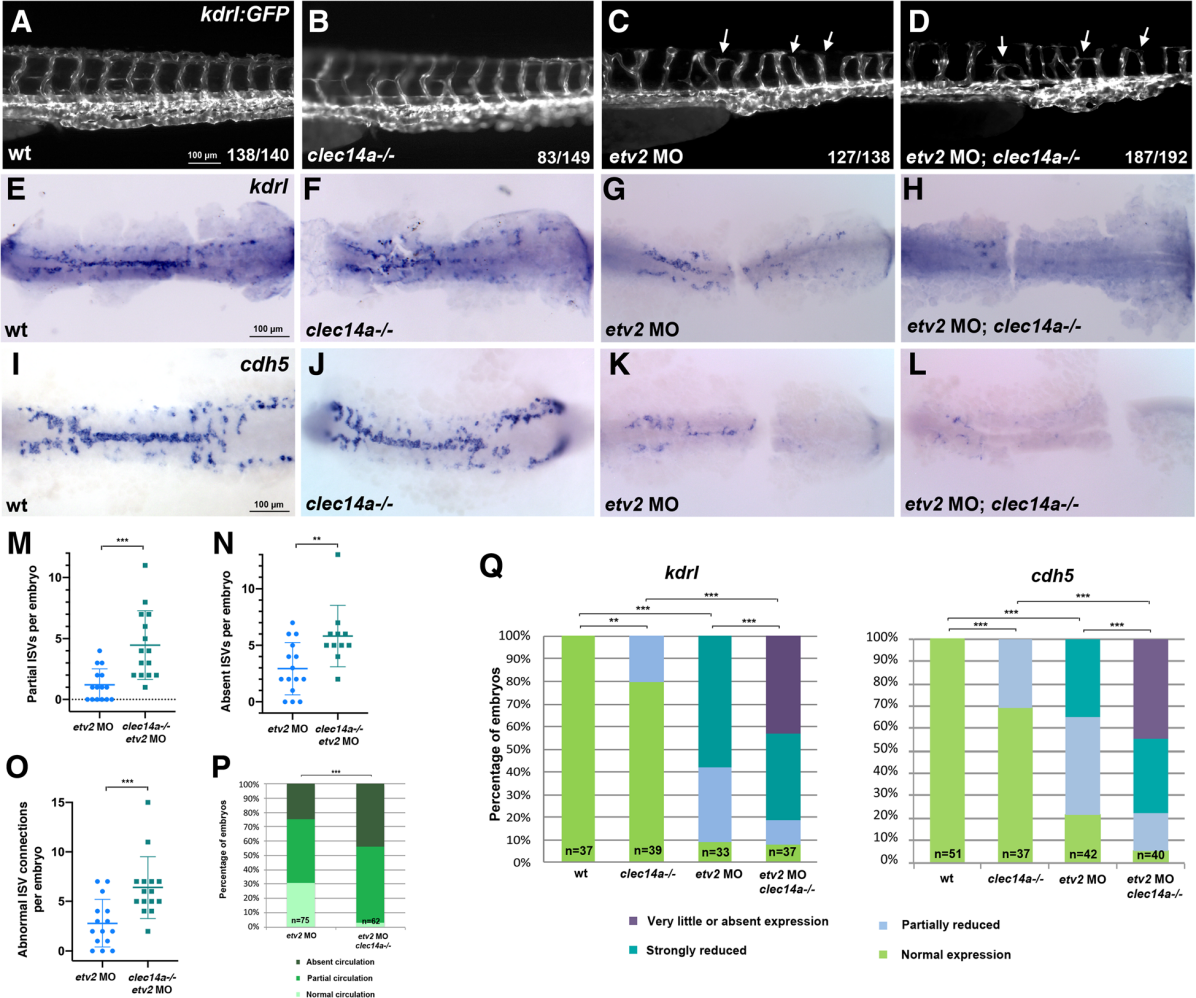
Combinatorial interaction between *etv2* and *clec14a.* **a**-**d** ISV sprouting defects (arrowheads) are increased in *clec14a* mutants injected with the low dose of *etv2* MO2 (0.25 ng) compared with *etv2* MO injection in wild-type embryos. *kdrl: GFP* transgenic embryos were imaged at 48 hpf; the trunk region is shown. **e**-**l** ISH analysis for *kdrl* (**e**-**h**) and *cdh5* expression at the 15–16-somite stages. Flat-mounted embryos; only the trunk and tail region is shown. Note a significant reduction in *cdh5* and *kdrl* expression in the embryos injected with low dose (0.125 ng) of *etv2* MO2. Much greater reduction is observed in *etv2* MO; *clec14a−/−* embryos. **m**-**o** The number of partial (**m**) or absent (**n**) ISVs per embryo and abnormal ISV connections per embryo (**o**) in wild-type *kdrl: GFP* or c*lec14a−/−; kdrl: GFP* embryos at 48 hpf which were injected with 0.25 ng of *etv2* MO. 12–15 embryos were analyzed for ISV defects. ***p < 0.001, t-Student’s test. **p** Percentage of embryos with affected blood circulation at 48 hpf. All wild-type control and *clec14a−/−* embryos had normal blood circulation (not shown). To calculate statistical significance, embryos were compared with normal or defective circulation (combined partial and absent circulation categories) using Fischer’s exact test, ***p < 0.001. **q** Percentage of embryos with normal or reduced *kdrl* and *cdh5* expression. Statistical significance was calculated for wild-type versus *clec14a−/−* and *etv2* MO embryos using normal and reduced expression categories (all categories with reduced expression were combined), and for *etv2 MO; clec14a−/−* embryos versus *clec14a−/−* and *etv2* MO embryos using very little or absent expression categories (normal, partially and strongly reduced expression categories were combined). ** *p* < 0.01, *** *p* < 0.001, Fisher’s exact test. Data were combined from two independent experiments


To test if *clec14a* may participate in early vasculogenesis, we analyzed its genetic interaction with the ETS transcription factor Etv2 / Etsrp, a key regulator of early vasculogenesis [9, 10]. Injection of low doses of 0.125 or 0.25 ng per embryo of the previously validated *etv2* morpholino [9] resulted in partial defects in vasculogenesis and angiogenic sprouting. ISV sprouting defects at 48 hpf were more severe in *clec14a−/−; kdrl: GFP* embryos injected with 0.25 ng of *etv2* MO compared to wild-type *kdrl: GFP* embryos injected with the same dose of *etv2* MO (Fig. 4a-d). The number of partial ISVs, absent ISVs and abnormal ISV connections per embryo was significantly increased in *clec14a−/−; etv2 MO* embryos compared with wild-type embryos injected with *etv2* MO (Fig. 4m-o; because the majority of *clec14a−/−* embryos do not show ISV defects, they were not included in this analysis). While 31% of wild-type *etv2* MO injected embryos had normal axial blood circulation at 48 hpf, only 3% of *clec14a−/−; etv2* MO embryos had normal circulation, while the rest showed partially or completely inhibited axial circulation (Fig. 4p, Additional file 2: Movie S1 and Additional file 3: Movie S2). To test if *clec14a−/−* contributed to early vasculogenesis, we analyzed *kdrl* and *cdh5* expression in vascular endothelial progenitors at the 15–16-somite stages. There was no apparent difference between the majority of *clec14a−/−* embryos and wild-type controls while a fraction of *clec14a* mutant embryos showed a slight reduction in *kdrl* and *cdh5* expression (Fig. 4e, f, i, j, q). Wild-type embryos injected with a low 0.125 ng dose of *etv2* MO showed significant inhibition in *kdrl* and *cdh5* expression (Fig. 4g, k, q). Injection of the same dose of *etv2* MO into *clec14a−/−* embryos resulted in a much stronger inhibition in expression of these markers. In most embryos, very few cells with weak *kdrl* and *cdh5* expression were present (Fig. 4h, l, q). Because the phenotype of this severity was observed only in the double *etv2* MO; *clec14a−/−* embryos, this indicates a synergistic effect between *etv2* MO knockdown and *clec14a* mutation. These results suggest that *clec14a* contributes to vasculogenesis and functions during the specification and differentiation of vascular endothelial progenitors.

**Additional file 2: Movie S1.** Blood circulation in wild-type embryos injected with low dose of etv2 MO (0.25 ng). Tail region is shown at 48 hpf, anterior is to the right. (MPG 14006 kb)



**Additional file 3: Movie S2.** Absent blood circulation in *clec14a−/−* embryos injected with low dose of etv2 MO (0.25 ng). Tail region is shown at 48 hpf, anterior is to the right. (MPG 9052 kb)


**Fig. 5:**
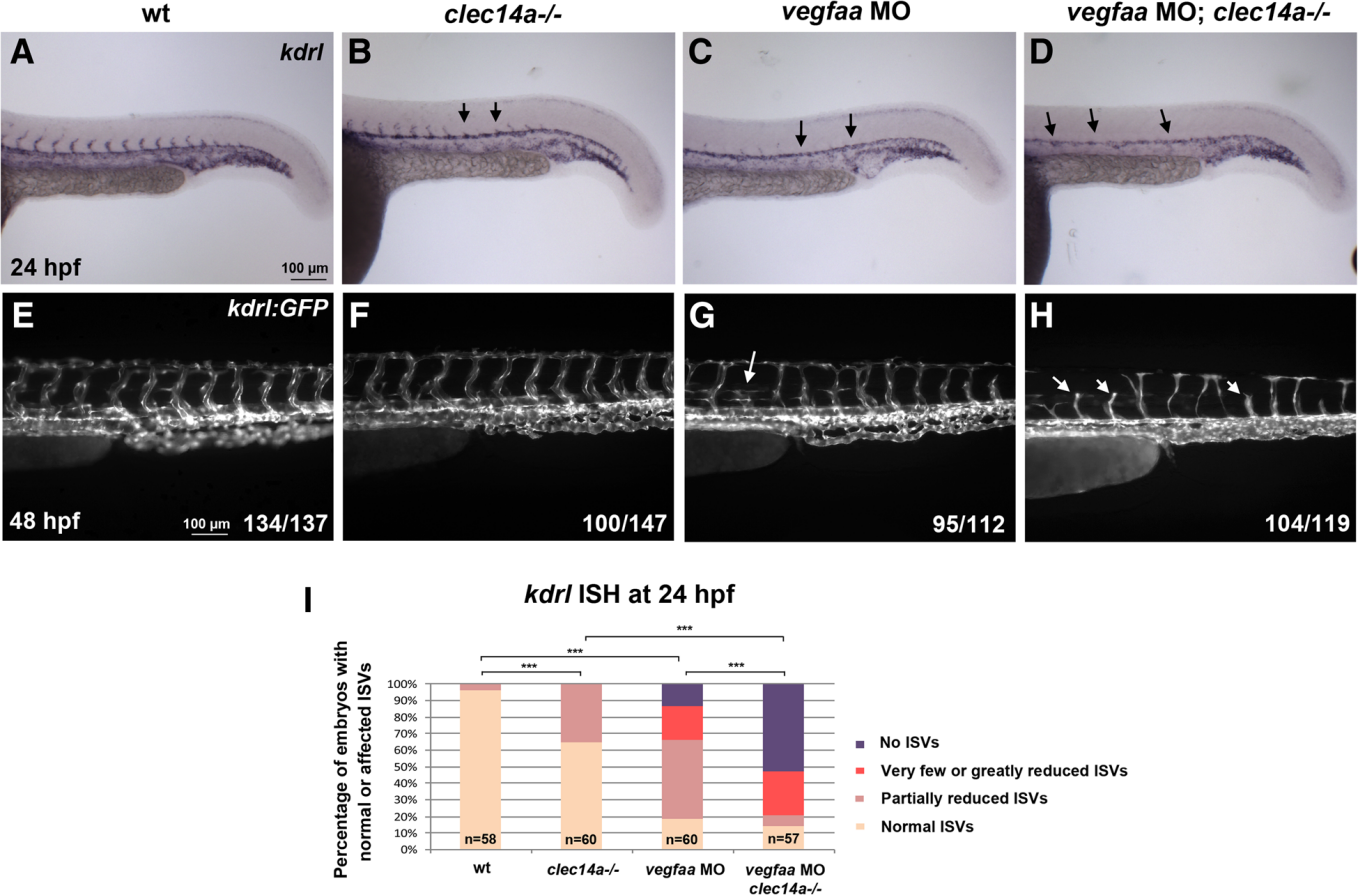
*clec14a* mutation and *vegfaa* MO knockdown show synergistic effect in inhibiting intersegmental vessel (ISV) sprouting. **a**-**d** ISH analysis of *kdrl* expression at 24 hpf. **e**-**h** Analysis of *kdrl: GFP* expression at 48 hpf. Note that partial inhibition of sprouting is observed using low dose of 2.5 ng *vegfaa* MO in wt embryos (**c**, **g**). The same dose in *clec14a* mutants results in a complete loss of sprouting at 24 hpf and significant inhibition of sprouting at 48 hpf (**d**, **h**). **i** Percentage of embryos with normal or inhibited ISVs based on *kdrl* expression at 24 hpf. *** *p* < 0.001, Fisher’s exact test; embryos with partially reduced ISVs were compared between wt and *clec14a−/−* or *vegfaa* MO groups; embryos with no ISVs were compared between the double knockdown *vegfaa* MO; *clec14a−/−* and the other groups. Data were combined from two independent experiments

We then tested genetic interaction between *clec14a* and Vegf signaling. As analyzed by *kdrl* expression at 24 hpf, low dose injection of the previously validated *vegfaa* MO [11] resulted in a partial reduction of ISV sprouting while axial vessel development appeared unaffected (Fig. 5c). Injection of *vegfaa* MO in *clec14a−/−* mutants resulted in a much stronger effect. The majority of embryos had no ISV sprouting at 24 hpf (Fig. 5a-d, i). More severe inhibition of ISV sprouting and DLAV formation was also apparent at 48 hpf based on *kdrl: GFP* expression in *vegfaa* MO; *clec14a−/−* embryos compared to single *clec14a* mutants and *vegfaa* MO embryos (Fig. 5e-h). Thus, inhibition of Clec14a and Vegfaa function results in a synergistic interaction.

## Discussion

Our results argue that *clec14a* participates in both vasculogenesis and angiogenesis in the zebrafish model system. *clec14a* mutant embryos display mild defects in angiogenesis. The defects are significantly more severe upon simultaneous depletion of *c1qr,* suggesting functional redundancy between the two genes. It was previously demonstrated that genetic mutants frequently exhibit upregulation of functionally homologous genes resulting in functional compensation [8]. Our results show that *c1qr* expression is upregulated in *clec14a* mutants. It is likely that *c1qr* can partially compensate for the absence of *clec14a* function, and therefore strong vascular defects are observed only when both homologs are inhibited. Similar redundant function between *clec14a* and *c1qr* in zebrafish embryos has been previously reported [5]. Differently from the previous study, we did not observe reduced survival among *clec14a* mutants. While the nature of *clec14a*^*ci15*^ allele is different from the previously described *c1qrl / clec14a* ^*cq30*^ allele [5], *clec14a*^*ci15*^ mutants show great reduction in *clec14a* mRNA expression likely due to the nonsense-mediated RNA decay, and therefore it is likely to be a null or close to null allele.

It is not clear why mouse and zebrafish *clec14a* mutants show quite different phenotypes. It is worth noting that previous studies have reported reduced angiogenesis upon Clec14a knockdown in vitro [2, 4], which is similar to the zebrafish *clec14a* mutant phenotype. Increased angiogenesis in mice was observed at much later stages (E13.5) [6] than the phenotypic analysis in ISV sprouting that we performed in zebrafish. It is possible that distinct vascular beds are affected differently by the loss of Clec14a function. The defects in ISV sprouting are quite mild, and are only pronounced upon combinatorial knockdown of *clec14a* and other genes involved in ISV sprouting. It is possible that Clec14a in mouse may also have an earlier role in promoting angiogenesis which is not apparent due to functional redundancy with C1qr or other related proteins.

Zebrafish *clec14a* expression was greatly downregulated or absent in *cloche/npas4l* or *etv2* mutants prior to 24 hpf [3, 9], while *etv2* expression was not affected in *clec14a/c1qrl*^*cq30*^ mutants [5], suggesting that *clec14a* functions downstream of *npas4l* and *etv2*. Intriguingly, *clec14a* mutants showed synergistic interaction with *etv2* during vasculogenesis. The combinatorial loss of *clec14a* and partial knockdown of *etv2* function resulted in a much greater reduction in vascular *kdrl* and *cdh5* expression than separate mutation or knockdown of *clec14a* and *etv2.* Synergistic interaction observed under partial *etv2* knockdown suggests that either *clec14a* functions downstream of *etv2* in the same pathway, or both *clec14a* and *etv2* function in two parallel and converging pathways during vasculogenesis. Yet despite the major defects in vasculogenesis at mid-somitogenesis stages, the same *clec14a−/−; etv2* MO knockdown embryos displayed relatively mild ISV sprouting defects at later stages. This partial recovery of vascular defects is likely due to the compensation of Etv2 deficiency by other ETS transcription factors such as Fli1b; we have previously demonstrated that Fli1b plays a major role in the partial recovery of defects in vasculogenesis observed in *etv2* mutant embryos [12].

Vegf signaling plays multiple roles during vasculogenesis and angiogenesis [13]. It has been recently shown that Clec14a physically interacts with Vegfr3 in vitro, and that mouse *Clec14a* knockout embryos show reduced *Vegfr3* expression and increased expression in *Vegfr2* which correlates with increased angiogenesis [6]. However, no increased angiogenesis was observed in zebrafish *clec14a* mutants. *flt4/vegfr3* and *kdrl* expression in the axial vasculature was not significantly affected in *clec14a−/−; c1qr* MO embryos, while ISV sprouting was reduced. It is possible that inhibition of angiogenesis may be a consequence of reduced Vegf signaling in *clec14a* mutants. Because Vegf signaling potentiates *etv2* expression during vasculogenesis [14], this would also explain synergistic interaction between the *etv2* and *clec14a* knockdown / mutation during vasculogenesis. However, synergistic interaction between *clec14a* mutation and *vegfaa* MO knockdown does not exclude a possibility that *clec14a* may also function in a separate parallel signaling pathway which promotes angiogenesis. Multiple other pathways including Angiopoietin-Tie2 and Delta-Notch signaling have been implicated in angiogenesis [15]. Further research will be required to test a potential *clec14a* involvement in these pathways.

## Conclusions

This study demonstrates the requirement for *clec14a* in both vasculogenesis and angiogenesis in the zebrafish model system. *clec14a* functions partially redundantly with a related protein C1qr / Cd93 during sprouting angiogenesis. In addition, *clec14a* genetically interacts with the ETS transcription factor *etv2* during vasculogenesis, demonstrating its novel role in promoting differentiation of vascular endothelial progenitors. Furthermore, our results show synergistic genetic interaction between *clec14a* and Vegf signaling. These results will promote our understanding of the mechanisms that guide vascular development.

## Methods

### Fish lines

*clec14a* ^*ci15*^ line was obtained by TALEN mutagenesis. TALENs were designed to the single *clec14a* exon using the TAL Effector Nucleotide Targeter software at https://tale-nt.cac.cornell.edu [16]. The target site TGTTCACTTGAACAAAAACTcattcacagatgcgcAGAATTATTGCAAACCTGGA was chosen near the 5′ end of the exon. The spacer sequence indicated in lowercase contains the HhaI enzyme recognition site CGCG. TALEN constructs were generated using Golden Gate assembly [17]. mRNA for the left and right TALEN arms was synthesized using T3 mMessage mMachine Kit (ThermoFisher). 50 pg each mRNA of was injected into 1-cell stage embryos. The following PCR primers flanking the TALEN recognition site were used to test for TALEN efficiency in pools of injected embryos: *clec14a_F* 5′-GCAGACATGGATTTCTGGATGG-3′, *clec14a_R* 5′-AGTGCTGTTGTCCACCGTC-3′. These primers amplify a 315 bp product that was fully digested by HhaI to produce 116 bp and 199 bp products in uninjected embryos. Retention of the 318 bp product in injected embryos indicated efficient TALEN mutagenesis. Adult carriers were identified by PCR genotyping using the same primers and HhaI enzyme digest. PCR products were sequenced using the *clec14a_F* primer to determine the deleted region.

Homozygous *clec14*^*ci15*^ embryos in *Tg (kdrl: GFP)*^*s843*^ [18] background and control Tg *(kdrl: GFP)* ^*s843*^ embryos were obtained from incrosses of homozygous *clec14*^*ci15*^*; kdrl: GFP* and wild-type *kdrl: GFP* adults, respectively. Embryos were raised at 28.5 °C or 32 °C temperature. Embryonic staging was performed according to the established criteria [19].

### Phenotypic analysis

Live embryos were analyzed at 28–72 hpf stages for vascular defects based on *kdrl: GFP* expression. Embryos which contained any ISVs in the trunk and tail region which were either not fully extended, absent or formed abnormal connections were counted as embryos with vascular defects.

### Morpholinos

A translation-blocking *c1qr* MO (GTCACTCTCATACTACTCGCTTTAG, Gene-Tools Inc), a 5-base *c1qr* mismatch MO (GTCAgTCTgATAgTACTCggTTTAG), a standard control MO (CCTCTTACCTCAGTTACAATTTATA, Gene-Tools Inc), a previously reported *vegfaa* MO (GTATCAAATAAACAACCAAGTTCAT) [11] and *etv2* MO2 (CACTGAGTCCTTATTTCACTATATC) [9] were used for experiments. All injections were performed at 1–2-cell stage.

### In situ hybridization

Whole mount in situ hybridization was performed using DIG-UTP labeled probes synthesized with T3, T7, or SP6 polymerase (Promega) as previously described [20]. Antisense RNA probes for the following genes were synthesized as previously described: *kdrl / flk1* [21], *fli1a* [21], *cdh5* [22], *cldn5b* [23], *flt4* [21], *clec14a / crl* [3].

### cDNA analysis and qPCR

RNA was purified from 15 to 20 wild-type and *clec14a* mutant embryos in *kdrl: GFP* background at 24 hpf using PureLink RNA purification kit (Thermo Fischer Scientific). cDNA was synthesized using SuperScript VILO cDNA synthesis kit (Thermo Fischer Scientific). PCR product corresponding to the coding sequence of *clec14a* was amplified with the following primers: TAAGCACTCGAGCACCATGGATTTCTGGATGGTATTACATC and TGCTTAAGATCTTTAGGTTTCCTCTTTTTCATTCACC. qPCR for *c1qr* expression was performing using the following primers: GCTTGACTCAGTTACCTGACGG and TTTCTGCTCGCTGTCCAACCC. Amplification was performed using the SYBR green PCR master mix and Step One Plus real-time PCR system (Thermo Fischer Scientific). Quantification was normalized to *ef1a* expression which was amplified using the following primers: TCACCCTGGGATGAAACAGC and ACTTGCAGGCCATGTGAGCAG. Two independent embryo replicates (15–20 embryos each) and 2–4 technical replicates for each sample were performed.

### Generation of c1qr:GFP reporter construct

939 bp *c1qr* upstream fragment was amplified by PCR from the CH211-202F3 BAC construct (obtained from Children’s Hospital Oakland Research Institute) using Expand High Fidelity PCR System (Sigma-Aldrich) and the following primers: *c1qr-F:* TCCATTTGCCTTCGGCTGGG and *c1qr-R*: CGAGTAGTATGAGAGTGACGGG. GFP-polyA sequence was PCR amplified from XGM2 (Xenopus EF1a-GFP-polyA construct) [24] using GFP-forward primer with an attached sequence that overlaps with the *c1qr* promoter fragment, and SP6 24-mer primers: GCGAGTAGTATGAGAGTGACGGGATGAGTAAAGGAGAAGAACTTTTCACTGG and CATACGATTTAGGTGACACTATAG. The final product was amplified by PCR using *c1qr-F* and SP6 24-mer primers and the initial two PCR products as DNA templates. PCR product was gel-purified, diluted in 1x Danieau buffer (58 mM NaCl, 0.7 mM KCl, 0.4 mM MgSO4, 0.6 mM Ca (NO3)_2_, 5 mM HEPES, pH 7.6) and injected into the embryo blastomere at 1-cell stage at a dose of 50 pg per embryo.

### Microscopy

Embryos were whole-mounted in 2% methylcellulose on glass slides. Images were captured using a 10x / NA 0.3 objective on an AxioImager Z1 (Zeiss) compound microscope with an Axiocam ICC3 color camera or Axiocam MMR grayscale camera (Zeiss). Images in multiple focal plans were captured individually and combined using the Extended Focus module within the Axiovision software (Zeiss). For ISH images at mid-somitogenesis stages, embryos were deyolked and flat-mounted in the araldite medium (EM Sciences).

## Additional files

Additional file 1 : **Figure S1.** *c1qr* MO inhibits reporter *c1qr:GFP* expression. (A) A diagram of the *c1qr* reporter construct. *c1qr* sequence of 939 bp immediately upstream of the initiating ATG codon which contains the *c1qr* MO binding site was fused with the GFP-polyA sequence using the fusion PCR approach. (B,C) GFP fluorescence in zebrafish embryos at the 22–24-somite stages which were injected with 50 ng of *c1qr:GFP* PCR product alone (B) or in combination with 10 ng of *c1qr* MO (C). Trunk and tail region is shown. Note that the distribution of injected DNA is typically highly mosaic. 11% of embryos injected with *c1qr:GFP* DNA showed multiple GFP+ cells in the tail region, while none of *c1qr:GFP* and *c1qr* MO co-injected embryos showed such expression (*p* = 0.01, Fischer’s exact test). Data were combined from two independent experiments. **Figure S2.** Injection of control MO (10 ng) does not enhance blood vessel defects in *clec14a* mutant embryos. (A-D) *kdrl: GFP* expression analysis at 48 hpf. (E) Percentage of embryos with vascular defects at 48 hpf. ***, *p* < 0.001; NS, not significant, Fischer’s exact test. Data were combined from two independent experiments. Error bars show standard error. **Figure S3.** Injection of 10 ng of a 5 base-pair *c1qr* mismatch MO does not cause additional vascular defects in wild-type or *clec14a* mutant embryos. (A-D) *kdrl: GFP* expression analysis at 48 hpf. (E) Percentage of embryos with vascular defects at 48 hpf. ***, *p* < 0.001; NS, not significant, Fischer’s exact test. Data were combined from two independent experiments. Error bars show standard error. (PDF 4910 kb)
