# Supplementary material for: Clec14a genetically interacts with Etv2 and Vegf signaling during vasculogenesis and angiogenesis in zebrafish
Source: BMC Dev Biol. 2019 Apr 5;19:6. doi: 10.1186/s12861-019-0188-6 (PMC6451255; doi:10.1186/s12861-019-0188-6)
Supplement: Supplementary file 3 — : Figure S1. c1qr MO inhibits reporter c1qr:GFP expression. (A) A diagram of the c1qr reporter construct. c1qr sequence of 939 bp immediately upstream of the initiating ATG codon which contains the c1qr MO binding site was fused with the GFP-polyA sequence using the fusion PCR approach. (B,C) GFP fluorescence in zebrafish embryos at the 22–24-somite stages which were injected with 50 ng of c1qr:GFP PCR product alone (B) or in combination with 10 ng of c1qr MO (C). Trunk and tail region is shown. Note that the distribution of injected DNA is typically highly mosaic. 11% of embryos injected with c1qr:GFP DNA showed multiple GFP+ cells in the tail region, while none of c1qr:GFP and c1qr MO co-injected embryos showed such expression (p = 0.01, Fischer’s exact test). Data were combined from two independent experiments. Figure S2. Injection of control MO (10 ng) does not enhance blood vessel defects in clec14a mutant embryos. (A-D) kdrl: GFP expression analysis at 48 hpf. (E) Percentage of embryos with vascular defects at 48 hpf. ***, p < 0.001; NS, not significant, Fischer’s exact test. Data were combined from two independent experiments. Error bars show standard error. Figure S3. Injection of 10 ng of a 5 base-pair c1qr mismatch MO does not cause additional vascular defects in wild-type or clec14a mutant embryos. (A-D) kdrl: GFP expression analysis at 48 hpf. (E) Percentage of embryos with vascular defects at 48 hpf. ***, p < 0.001; NS, not significant, Fischer’s exact test. Data were combined from two independent experiments. Error bars show standard error. (PDF 4910 kb) [file 12861_2019_188_MOESM1_ESM.pdf]

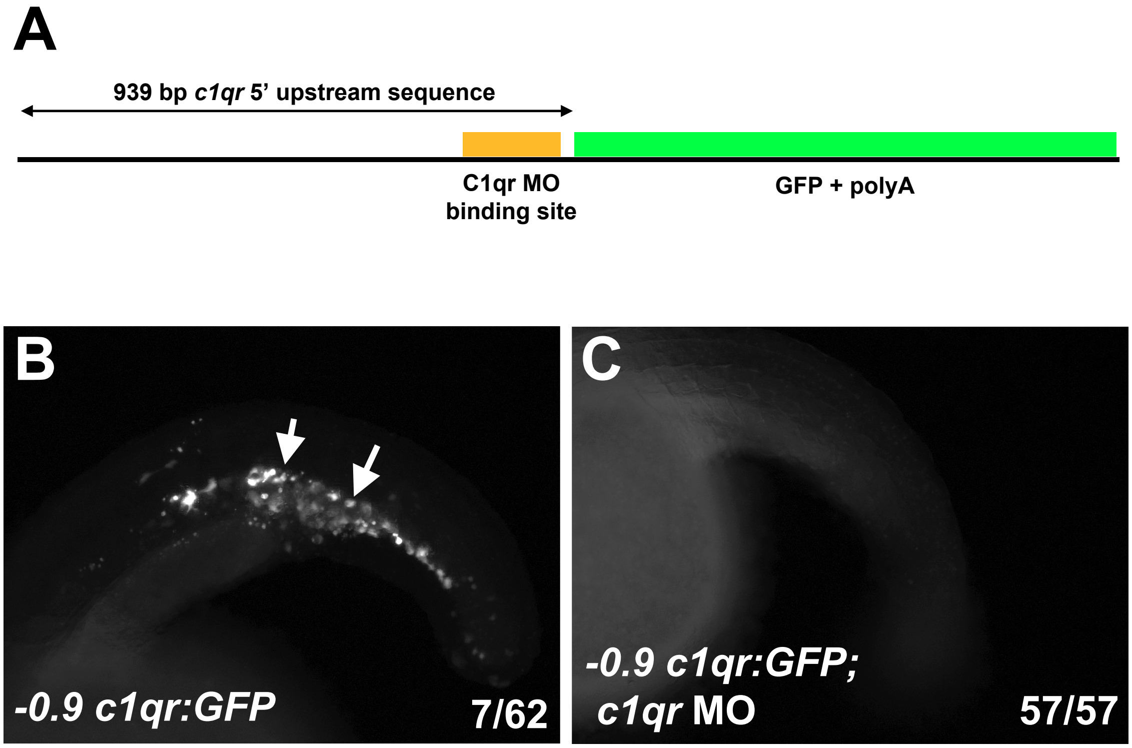

Figure S1. *c1qr* MO inhibits reporter *c1qr:GFP* expression. (A) A diagram of the *c1qr* reporter construct. *c1qr* sequence of 939 bp immediately upstream of the initiating ATG codon which contains the *c1qr* MO binding site was fused with the GFP-polyA sequence using the fusion PCR approach. (B,C) GFP fluorescence in zebrafish embryos at the 22-24-somite stages which were injected with 50 ng of *c1qr:GFP* PCR product alone (B) or in combination with 10 ng of *c1qr* MO (C). Trunk and tail region is shown. Note that the distribution of injected DNA is typically highly mosaic. 11% of embryos injected with *c1qr:GFP* DNA showed multiple GFP+ cells in the tail region, while none of *c1qr:GFP* and *c1qr* MO co-injected embryos showed such expression (p=0.01, Fischer's exact test). Data were combined from two independent experiments.

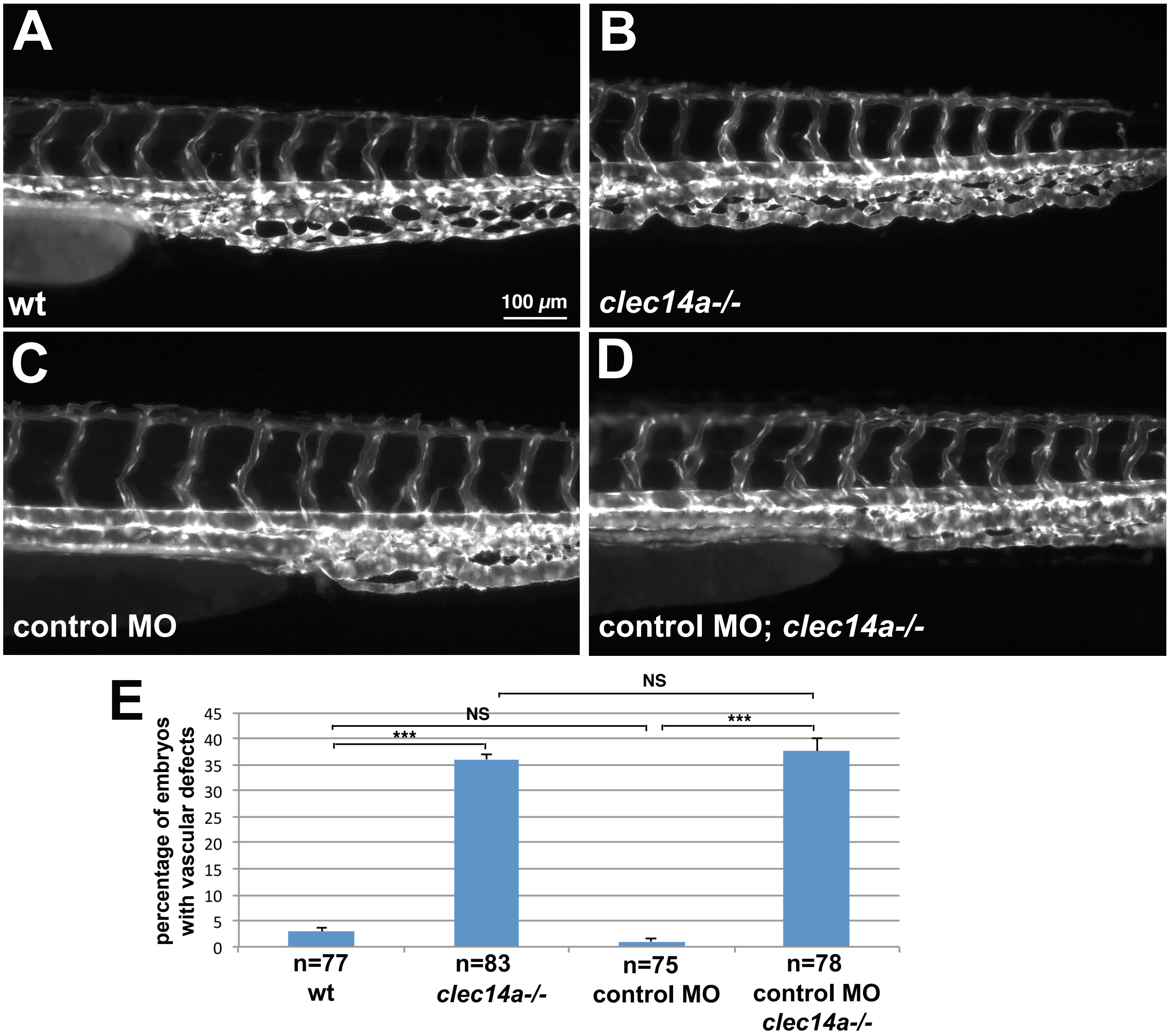

Figure S2. Injection of control MO (10 ng) does not enhance blood vessel defects in *clec14a* mutant embryos. (A-D) *kdrl:GFP* expression analysis at 48 hpf. (E) Percentage of embryos with vascular defects at 48 hpf. ***, $p<0.001$; NS, not significant, Fischer's exact test. Data were combined from two independent experiments. Error bars show standard error.

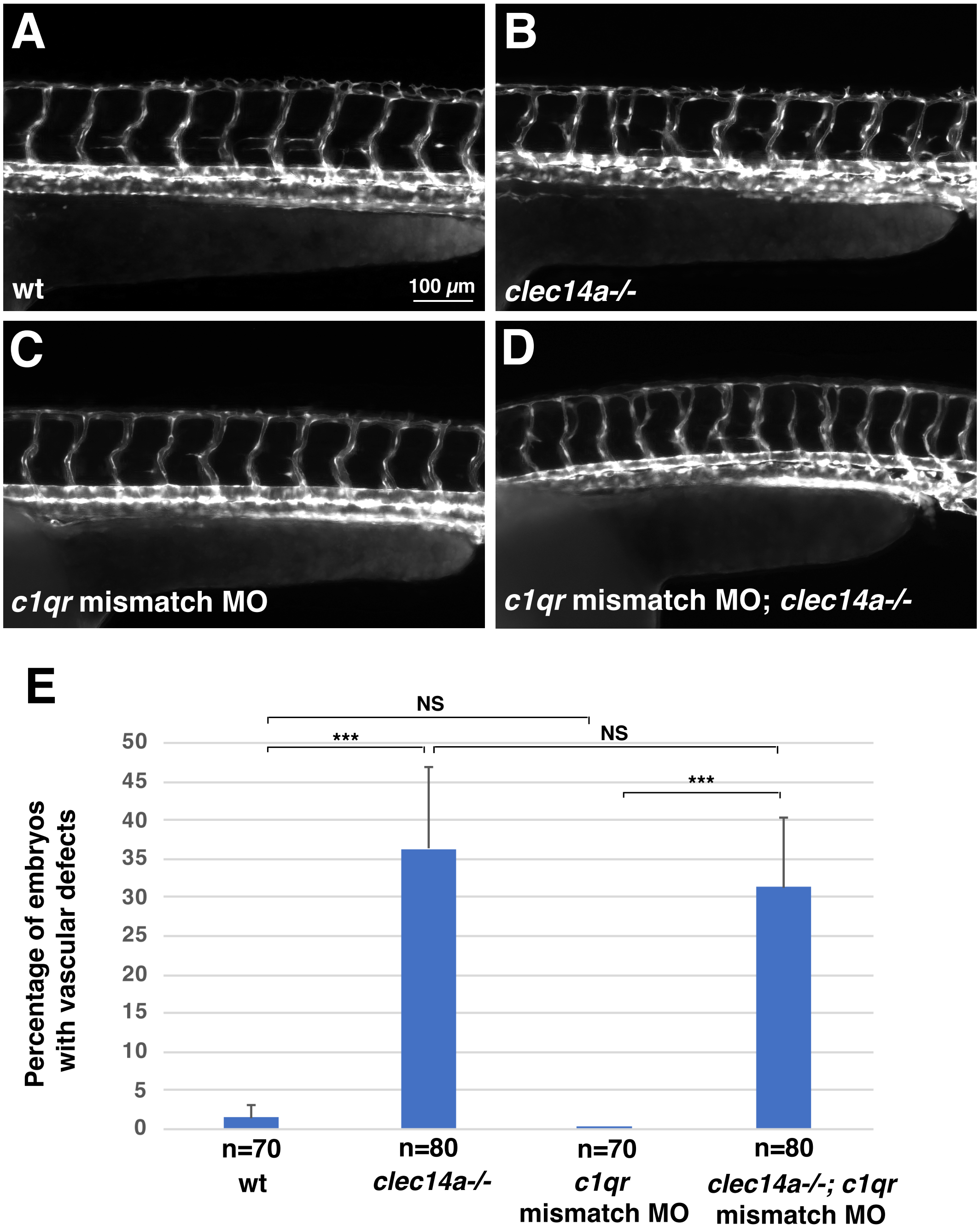

Figure S3. Injection of 10 ng of a 5 base-pair *c1qr* mismatch MO does not cause additional vascular defects in wild-type or *clec14a* mutant embryos. (A-D) *kdrl:GFP* expression analysis at 48 hpf. (E) Percentage of embryos with vascular defects at 48 hpf. ***$p<0.001$; NS, not significant, Fischer's exact test. Data were combined from two independent experiments. Error bars show standard error.
